# Mouse modeling for anxiety disorders in older adults

**DOI:** 10.31491/apt.2021.09.067

**Published:** 2021-09-30

**Authors:** Martin Darvas, Nadia Postupna, Warren Ladiges

**Affiliations:** a Department of Laboratory Medicine and Pathology, School of Medicine, University of Washington, Seattle, WA 98195, USA.; b Department of Comparative Medicine, School of Medicine, University of Washington, Seattle, WA 98195, USA.

## Abstract

Anxiety disorders are common in older adults and are strongly associated with increased risk for numerous age-related conditions. Preclinical mechanistic data are needed to identify more specific therapeutic targets for treating and preventing these disorders. Mice serve as excellent preclinical models as they have been used extensively in aging studies, and behavioral tests have been developed. A panel of tests would capture the important clinical aspects of apathy, anxiety, and psychomotor behavior and allow longitudinal testing strategies in a rigorous and minimally stressful manner.

Anxiety disorders in older adults are associated with an increased physical disability, increased risk for mild cognitive impairment and dementia [1]. Current estimates of the prevalence of anxiety disorders in US older adults suggest a 1-year prevalence of close to 12 percent [2]. Anxiety and depressive disorders are often present as comorbid conditions with strongly increased risks for the onset of cardiovascular diseases, diabetes, stroke, obesity, and cognitive decline [3, 4]. Indeed, anxiety and depressive disorders are often part of non-cognitive neuropsychiatric symptoms found in dementia, and as such are associated with high levels of distress in both dementia patients and caregivers [5, 6]. Depression is among the most common behavioral and psychological signs of dementia and can be an early or preclinical indicator. Alzheimer’s disease (AD) is the major cause of dementia and it has been shown that a clinical diagnosis of depression in both early and late-life increases the risk for AD. Hence, depressive and anxiety disorders need to be considered as important risk factors for a multitude of aging-related conditions.

A majority of the reported studies on anxiety disorders and aging have been correlative in nature. Preclinical mechanistic data are needed to identify more specific therapeutic targets for treating and preventing these disorders associated with aging. There is no ideal animal model for this purpose, but rodents have traditionally been used. Laboratory rats are in general more responsive to behavioral testing than laboratory mice. However, mice have a much more extensive background in aging studies, and behavioral tests have been developed and validated for a number of mouse strains. The problem is that they are not well standardized so that it is challenging to replicate studies from lab to lab. A panel of tests is needed to identify the various phenotypes associated with anxiety, but a generally accepted panel has not been developed for this purpose. Several behavior tests have been demonstrated to capture the important clinical symptoms of apathy, anxiety, and psychomotor behavior. Here, we focus on testing procedures that allow longitudinal testing strategies. Hence, tests that require aversive experiences such as social isolation, food restriction, surgical interventions, or exposure to noxious stimuli like forced swimming or foot shocks are not included. Some of these excluded tests, like self-stimulation procedures, may have excellent validity for measuring domains like anhedonia (loss of interest in pleasurable activities) or motivation, yet they contain strong learning components and do lend themselves readily to longitudinal study designs. Other concerns with well- established procedures for behavioral despair like the forced-swim test are not recommended because of the rather frail health condition of geriatric mice.

Based on published studies and our experience, we suggest the following conditions be included in a panel for mouse models of anxiety disorders. The first is apathy. Apathy has been defined as a reduction in goal-directed behavior [7]. Many established procedures to measure goal-directed behavior in mice mostly involve strong elements of learning and are therefore difficult to integrate into longitudinal experimental designs. Nest building, self-grooming, and burrowing are goal-directed innate behaviors that correspond to daily abilities which are often disrupted in anxiety disorders and neurodegenerative diseases. These procedures have been tested in rodent models of apathy [8] and can be performed repeatedly in longitudinal study designs. Nest building is measured using Deacon’s comprehensive five-point rating scale [9]. Self-grooming can be assessed with the splash test. For this test, a 10 percent sucrose solution is squirted on the dorsal coat of the mice in their home cage, followed by a recording of the grooming frequency during 5 minutes immediately after sucrose splashing [10]. In the burrowing test, mice spontaneously empty a tube filled with food pellets as described by Deacon [11]. Lower scores for the nest-building rating scale, reduced grooming frequency in the splash test, and reduced burrowing are indicators of apathy and impaired activities of daily living.

Secondly, the novelty-suppressed feeding test can be used to measure anhedonia, a depression-like behavior [12]. Mice are food-deprived for 24 hours with free access to water and then placed into the corner of a novel open-field apparatus that has a food pellet placed in the center. The latencies to approach and then eat the pellet are recorded. Increased latencies are taken as indicators for anhedonia. Third, anxiety can be measured by the open-field and elevated-zero maze tests [13]. The movement of the mice in either maze is video-recorded and analyzed with video-tracking software. Time spent in the center of the open-field arena over a period of 30 minutes and time spent in the open areas of the elevated-zero maze over a period of 5 minutes is calculated as measures of anxiety [14]. Fourth, psychomotor disturbances can be assessed by the open-field test. The overall distance traveled by mice over a 30-minute testing period is measured and analyzed as indicators of overall locomotor agitation or retardation. Lastly, since attention and working memory are impaired in anxiety and depressive disorders, the Y-maze procedure is excellent for testing cognitive abilities. As with the other tests described above, the Y-maze can be used longitudinally without skewing results with repeated testing.

In conclusion, mouse modeling for age-related anxiety disorders can be successful if rigorous and minimally stressful test procedures are used in combination as a panel format.
